# Supplementary material for: A Comprehensive Self-Resistance Gene Database for Natural-Product Discovery with an Application to Marine Bacterial Genome Mining
Source: Int J Mol Sci. 2023 Aug 4;24(15):12446. doi: 10.3390/ijms241512446 (PMC10419868; doi:10.3390/ijms241512446)
Supplement: Supplementary file 1 [file ijms-24-12446-s001.zip › Figure S1. MarRef BGCs classified based on phylum origin.pdf]

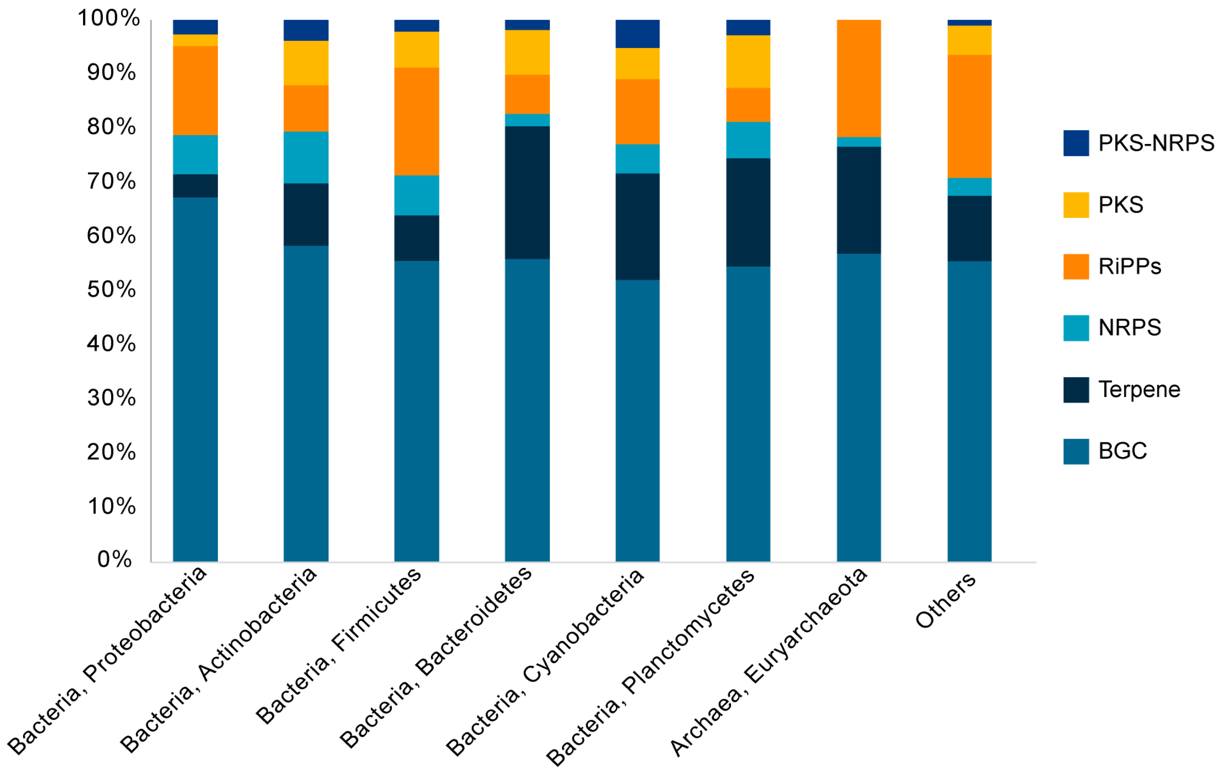

Figure S1. Comparison of the different types of BGCs produced by typical bacterial phyla in MarRef.
